# Buffalo Academy of Medicine

**Published:** 1911-07

**Authors:** 


### SOCIETY PROCEEDINGS

### Buffalo Academy of Medicine

THE annual meeting and election of the Buffalo Academy of Medicine was held in the academy rooms, Public Library building, Tuesday evening, June 13.

The meeting was called to order by Dr. L. Kauffman, who has been acting president since the death of Dr. Carlton C. Frederick. Dr. Kauffman was chairman of the section on medicine during the past year.

The reports of the officers showed the Academy to be in a very prosperous condition with a comfortable balance in the banks.

Dr. Julius Ullman presented the annual report for the committee on the Contagious Hospital. Dr. P. W. Van Peyma presented the annual report of the Committee on School Inspection. Dr. L. Kauffman presented the annual report of the Committee on Epidemic Poliomyelitis.

The tellers, Wm. F. Jacobs and Douglass P. Arnold, reported the following result of the election: president, Stephen Y. Howell; secretary, Harry R. Trick; treasurer, Lawrence Hendee; trustee for three years, John H. Pryor.

A collation, largely attended and marked by every evidence of good fellowship, was served at the close of the meeting.

